# Does treatment rate impact the efficacy of extracorporeal shock wave lithotripsy for kidney or ureteral stones?

**Published:** 2007

**Authors:** K. Muruganandham, Aneesh Srivastava

**Affiliations:** Department of Urology and Renal Transplantation, Sanjay Gandhi Post Graduate Institute of Medical Sciences, Lucknow, India. E-mail: anand78_uro@yahoo.co.in

## SUMMARY

In a retrospective study Chacko, Moore *et al* have examined the impact of treatment rate on the efficacy of extracorporeal shock wave lithotripsy (ESWL) for solitary stones less than 2 cm located in the kidney and stones in the proximal ureter, with a stone surface area (SSA) between 30 and 90 mm^2^. Confounding variables like stone location, stone radio-opacity, anesthesia type, stone size and machine type were controlled. From May 2002 to August 2004, 439 patients underwent ESWL for a solitary kidney stone between 1 and 2 cm or for stones with an SSA of 30 to 90 mm^2^ located in the kidney or proximal ureter. Adequate follow-up was available for 349 patients (80%) of which 135 had renal stones between 1 and 2 cm, 137 had renal stones less than 1 cm (SSA between 30 and 90 mm^2^) and 77 had a proximal ureteral stone with an SSA of between 30 and 90 mm^2^. Patients were subjected to ESWL using either Fast Rate (120 shocks/min, FR) or Slow Rate (between 70 and 80 shocks/min, SR). Parameters that were studied were the total number of shock waves, power index, fluoroscopy time, treatment time and stone-free rate (SFR).

Stone characteristics and SSA were similar in the two groups for all three stone categories. About 40% of stones between 1 and 2 cm and 52% of stones between 30 and 90 mm^2^ were in the lower pole. There were no statistical differences between the FR and SR groups with respect to stone location. The average number of shocks and power indices in the FR group were significantly higher compared to the SR group for each stone category (*P*=0.05). Average fluoroscopy time in the FR group was less for renal stones but more for ureteral stones compared to the SR group but the difference was not statistically significant. The ESWL treatment time was slightly less in the FR group across all stone categories compared to that in the SR group but only statistically significant for renal stones between 30 to 90 mm^2^. Slower treatment rate resulted in improved SFRs compared to the faster rate. Difference in the SFR was only statistically significant for the 1 to 2 cm renal stone category. For renal stones between 1 and 2 cm the SFR was 67% in the SR group but 46% in the FR group (*P*=0.05). There were no differences in outcome based on renal stone location.[1]

## COMMENTS

This study is a retrospective study in which it is difficult to have comparable groups. Lesser radio-opaque stones and ureteric stones >90 mm^2^ were excluded from the study. Parameters like BMI of patient, calyceal anatomy, stone composition and stone margin (smooth vs. spiked) were not studied. The grade of the urologist, postESWL complications and the type of ancillary treatment required were also not included in the analysis. In this study there was no difference in SFR between lower polar and nonlower polar stones which is in contradiction to the published results. Also, the physical basis for the improved results with slower rate of ESWL was not explained adequately.

It has been suggested by many authors that the ESWL treatment rate may independently impact its efficacy. *In vitro* studies using stone phantoms have shown variable stone fragmentation rates based on treatment rates. Vallancien *et al* suggested that the treatment rate for best stone fragmentation was 75 or 150 shocks/min.[2] However, recent *in vitro* and animal studies using the electrohydraulic lithotriptors suggest that the most efficacious treatment rate is 30 or 60 shocks/min.[3] The optimal treatment rate at which ESWL should be performed for a particular renal or ureteral stone remains to be defined.

In an *in vitro* study Greenstein *et al* compared shock wave rates of 30, 60, 90, 120 and 150 shocks/min using ceramic stones.

The most effective rate for stone fragmentation was 60 shocks/min with no significant difference between 30 and 60 shocks/min.[4] Weir *et al* examined the number of shocks required to fragment solid plaster stones at treatment frequencies of 60 to 117 shocks/min and found that the fewest shocks required to fragment the stones were 60 shocks/min.[3] Paterson *et al* placed stones percutaneously in porcine kidneys and compared the results of 30 and 120 shocks/min.

Results showed significantly more complete fragmentation at 30 rather than 120 shocks/min.[5]

Madbouly *et al* performed a prospective, randomized study in 156 patients assigned to treatment groups of 60 and 120 shocks/min.

Using multivariate logistic regression analysis they reported less number of shock waves, higher treatment time, higher SFR with 60 shocks/min. However, in this study they did not control stone characteristics or the number of ESWL treatments.

The ESWL efficacy was significantly better at a slower treatment rate (70 to 80 shocks/ min) than at a more rapid rate (120 shocks/min).[6]

In a prospective randomized study Yilmax and Batislam *et al* compared three different shock wave frequencies in170 patients with radio-opaque kidney stones. Group 1 (n=56) received 120 shock waves/min, Group 2 (n=57) received 90 shock waves/min and Group 3 (n=57) received 60 shock waves/min. The successful therapy rate in Groups 2 and 3 was prominently greater compared with that for Group 1 and the difference was statistically significant (*P* = 0.032 between Groups 1 and 2 and *P* = 0.015 between Groups 1 and 3). The analgesic or sedative requirement in Groups 2 and 3 was lower than that in Group 1 and the difference was statistically significant (*P* = 0.003 between Groups 1 and 2 and *P* = 0.001 between Groups 1 and 3). The treatment duration was longer in Group 3 than in Groups 1 and 2 and the difference was statistically significant (*P* = 0.000 between Groups 1 and 3 and *P* = 0.009 between Groups 2 and 3).[7]

The existing literature appears to support the use of slower rate during ESWL therapy with a tilt towards an average of 60 shocks/min. However, well-designed studies with more number of patients may be required to support or contradict this finding. Slower Rate has the potential to accomplish better ESWL efficacy with fewer shock waves and a lower power index; thus minimizing the extent of shock wave-induced renal injury.
